# Supplementary material for: Food and nutrient intake among non-pregnant, non-lactating women of reproductive age of Mbeya in Tanzania: A repeated 24-hour dietary recall
Source: PLOS Glob Public Health. 2024 Dec 5;4(12):e0004010. doi: 10.1371/journal.pgph.0004010 (PMC11620714; doi:10.1371/journal.pgph.0004010)
Supplement: Attachment — Submitted filename: Review, Food and nutrients intake_ FINAL.docx [file pgph.0004010.s002.docx]

**Food and nutrient intake among non-pregnant, non-lactating women of reproductive age in Tanzania: A repeated 24-hour dietary recall**

**Authors:**

Nyamizi J. Ngassa^1^, Ray M. Masumo^1*^, Adam Hancy^1^, Esther J. Kabula^1^, Erick Killel^1^, Jackline Nusurupia^1^, Adeline Munuo^1^, Hoyce Mshida^1^, Rose Mpembeni^2^, Elizabeth lyimo^1^, Rose V. Msaki^1^, Theresia Jumbe^3^, Germana H. Leyna^1, 2^

**Affiliations:**

1. Tanzania Food and Nutrition Centre (TFNC), Dar es Salaam, Tanzania
2. Muhimbili University of Health and Allied Sciences (MUHAS), Dar es Salaam, Tanzania
3. Sokoine University of Agriculture (SUA), Morogoro, Tanzania

*Corresponding author

E-mail: [rmasumo@yahoo.com](mailto:rmasumo@yahoo.com)

**Abstract**

A study was conducted among 500 women of reproductive age who were not pregnant or lactating, residing in seven districts of the Mbeya region. The study estimated the dietary intake of these women using a 24-hour dietary recall survey. The SAS macros (version 9.4) were used to analyse the quantities consumed and explore the usual intakes of three fortified food vehicles (oil, wheat flour, and sugar), energy and protein, and 12 micronutrients (vitamins and minerals) of interest. The results showed that the median intake of oil, wheat flour, and calories was 36.47g, 110.53g, and 2,169.9 kcal, respectively. The median protein intake was 63.5g, higher than the recommended value of 56.0g. Among the micronutrients, zinc had the highest prevalence of inadequate intake (91.2%), followed by iron (82.2%), and vitamin B12 (80.0%). Vitamin E had the highest nutrient gap (50.7%), while the nutrient gap in Q1 was relatively low (9.8%). There was a moderate prevalence of inadequate intake of vitamin C (46.5%) and riboflavin (54.8%), with a nutrient gap in Q1 (42%). Inadequate intake of vitamin A, thiamine, niacin, vitamin B6, and folate was moderate, ranging from 32.6% to 44.4%, with a nutrient gap at Q1 ranging from 16.2% to 34%. Our study has shown a highly prevalent of inadequate intake of micronutrients among non-pregnant and non-lactating women of reproductive age in Mbeya region, Tanzania. The findings highlight the need for increased attention towards the intake of vitamin E, Zinc, iron, and vitamin B12, followed by riboflavin and vitamin C. Moreover, our study suggests that the prevalence of inadequate intake of vitamin A can be significantly reduced among women given the usual intake of oil. In this context, oil emerges as a promising candidate for fortification. However, fortified wheat flour would have a limited impact on the nutrient gaps observed due to its limited reach among non-pregnant and non-lactant WRA in Mbeya region Tanzania.

**Key words**: 24-hour dietary recall; women of reproductive age; micronutrients; energy and protein; fortified food

**Introduction**

Micronutrient deficiencies can have adverse effects on the health of women of reproductive age (WRA), their pregnancy outcomes, growth, and the development of their offspring [1]. WRA are particularly susceptible to micronutrient deficiencies due to inadequate dietary intake, unequal distribution of food within the household, societal norms, and gender-based discrimination that expects women to prioritize their family's nutritional needs over their own [2].

It is estimated that around three billion people worldwide cannot afford to maintain a healthy diet, as reported in 2021[3]. The consumption of low-micronutrient diets is a significant contributor to micronutrient deficiencies, which persist as a major challenge in developing countries such as Tanzania [4]. Micronutrient deficiencies, particularly with regards to iron, vitamin A, iodine, and zinc, are among the top ten leading causes of morbidity and mortality among women of reproductive age in low-income countries [5,6]. These deficiencies can have long-term consequences on health, learning ability, and productivity. Iron Deficiency Anaemia (IDA), Iodine Deficiency Disorders (IDD), and Vitamin A Deficiency (VAD) are the most common micronutrient deficiencies reported in Tanzania, according to the Tanzania Demographic and Health Survey (TDHS) in 2015-16 [7]. Other micronutrient deficiencies of concern in Tanzania include Zinc, Folic Acid, Calcium, and some B vitamins (B12 and niacin), as reported by the TDHS in 2010 [8]. In Tanzania, the prevalence of anaemia among women of reproductive age (15-49) was reported to be 45% with pregnant women having a much higher prevalence (57%) [9,10]. Furthermore, the TDHS 2010 reported that 36% of women of reproductive age (15-49 years) in Tanzania had Vitamin A deficiency. Although the role of Vitamin A deficiency in causing anaemia is not fully established, it may affect red blood cell production [11].

Women of reproductive age (WRA) make up 48% of the total female population in Tanzania, and the total fertility of 4.39 [7,12]. Energy and protein shortages, along with micronutrient deficiencies, are common among non-pregnant and lactating women, posing serious public health concerns [1]. In Africa, literatures have shown that dietary behaviors are influenced by cultural, geographical, and food preferences [13-18]. Additionally, women from socially and economically disadvantaged backgrounds are more affected by these issues [14-19]. Due to the limited coverage and longer periodicity of national nutritional surveillance in the country, ad hoc surveys offer a quick way to evaluate the diets of targeted populations.

There is a lack of information about the food and nutrient intake in most sub-Saharan African countries, including Tanzania [6,13,15,19]. This information is important to understand energy intake, as well as the intake of macronutrients (fats, proteins, and carbohydrates), micronutrients (selected vitamins and minerals), food groups, and food fortification. With this knowledge, policies and programs can be informed with new evidence to improve nutrient intake, dietary patterns, and the overall nutritional status of the population [6,13,15,19]. Therefore, the present study aimed to estimate and describe the distribution of food and nutrient intake among women of reproductive age 15–49 years who are non-pregnant or lactating in seven districts of the Mbeya region in Tanzania.

**Materials and Methods**

***Ethical statement***

This study obtained ethical permit from the National Health Research Ethics Review Committee (NatHREC) at National Institute for Medical Research with reference no. NIMR/HQ/R.8a/Vol.IX/3964. All study participants provided written consent prior to participating in the study and, participants did not receive financial compensation for participating. All data were anonymised and, will be destroyed when the project is concluded.

***Study design and study population***

A cross-sectional study was conducted in the Mbeya region of Tanzania from July to September 2022. The study enrolled non-pregnant, non-lactating women aged 15 to 49 years old from all seven districts of the region, namely Chunya DC, Mbeya DC, Mbeya City, Mbarali DC, Kyela DC, Rungwe DC, and Busokelo DC.

***Sample size and sampling procedures***

To conduct a statistically valid study, a pre-calculated sample size of 500 non-pregnant, non-lactating women of reproductive age (15 to 49 years) has been estimated. This estimation takes into account a significance level of 5%, a 95% confidence interval, and a design factor of 2. An additional 10% of the sample size has been added to account for those who refused to participate or were unable to communicate due to illness. However, residents in refugee camps have been excluded from the study based on the Lwanga and Lemeshow formula to ensure that the minimum sample size needed to obtain statistically valid results is met [20].

To select participants for the study, a two-stage sampling method was used. In the first stage, three enumeration areas (EAs) were chosen for each district using the probability proportional to size (PPS) method. In the second stage, 22 eligible households were randomly selected from each chosen EA. This was based on a complete list of all households within the EA. Before data collection began, the study team worked with local leaders to list all eligible households in the area. In each selected household, only one woman not pregnant and lactating aged between 15 and 49 was chosen for the survey. As part of the study, a sub-sample of 100 (20%) participants was selected for a 24-hour dietary recall [21-23]. To ensure that the data on consumption was not affected by leftovers or seasonality, about five participants were randomly selected for a second recall between 3 to 8 days after the first recall from each EA [21-23].

***Data Collection***

We collected data using a household questionnaire, women's questionnaire, 24-hour dietary recall questionnaire, and anthropometry form. The household module obtained demographic information for each respondent and their household. We administered a 24-hour dietary recall questionnaire to assess information on foods and drinks consumed. We used this to inform the potential contributions of industrially fortifiable foods to reduce micronutrient deficiencies and estimate the percentage of the population consuming selected fortifiable food vehicles. We used a tablet-based questionnaire programmed into the Open Data Kit via the Kobo toolbox. Paper forms were also available. The survey was translated from English to Swahili and back-translated for accuracy by different translators.

***Data cleaning and preparation***

We extracted 24-hour recall data from the Kobo toolbox and imported it into Excel and Stata for cleaning and analysis. We used recipe name, method, ingredients, and raw food quantities to update the list. We also recorded amounts of cooked recipes consumed and leftover in local or conventional units. The researchers in the study converted the portion sizes of food consumed into grams by multiplying the portion consumed by the measured weights of the foods listed in the photobook created for the study [21]. If the food wasn't listed in the photobook, they multiplied the reported volume of consumption by density values published by the Food and Agriculture Organization (FAO) and the United States Department of Agriculture (USDA). They also calculated the differences between the amount served and leftovers for a more accurate measurement.

Descriptive statistics and the EXPLORER command in the Statistical Package for the Social Sciences (SPSS) syntax were used to identify outliers. We employed various techniques to address extreme values, such as measuring certain food samples like a tablespoon of oil, a level, rounded, or heaped teaspoon of sugar or salt, and a gram of small, medium, and large tomatoes. Additionally, we cross-checked the amounts from different sources, including the Tanzania Food Composition Table (TFCT) recipe, Google searches, and interviews with restaurant and catering chefs to confirm ingredient ratios in recipes and common cooking and preparation methods.

***Food matching, data quality control and validation***

For the 66 distinct single foods extracted from recipes, we calculated the ingredient fraction for each recipe ingredient by multiplying the ingredient ratio with the corresponding USDA yield factor and the FAO density data based on the cooking or preparation method. This allowed us to estimate the consumed quantity of the cooked single food, by applying this calculated ingredient fraction to the reported total amount of the recipe consumed. We matched the 71 single foods directly reported (e.g., avocado, rice eaten raw, or sugar) with items included in the FCT on a 1:1 basis, and the quantities reported in the survey were taken as the quantities consumed. We developed a Visual Basic for Applications (VBA) Excel macro to automate the finding of each reported single food name and compute its corresponding consumed quantity. These single foods were then matched with four food composition tables, two for Tanzania (one with non-fortified oil and wheat flour and one with fortified values), and two for Uganda (one without and one with fortified values), to determine their nutrient content. We matched new foods not documented in the TFCT with food composition data from Kenya, Malawi, or the United States as appropriate. We selected the closest matching for foods (e.g., for region-specific birds and fish). A comparison of the total nutrient intake with the harmonized average requirement (H-AR) for each nutrient, except for energy, carbohydrate, and dietary fibre where the national values were used as reference based on the age group. We defined the inadequacy of intake if the total nutrient intake was below the reference values. **Table 1** presents the H-AR and the national reference values.

***Data Analysis***

We calculated the amount consumed, in grams, of fortification vehicles such as oil, wheat flour, sugar from homemade recipes, and sugar from industrially made foods or beverages. If we didn't have information on a particular fortification vehicle, we set its value to zero. Since we did not have data on the specific brands of fortification vehicles, we used the fortificant values in the TFCT and Uganda Food Composition Table (UFCT) to estimate the prevalence of inadequate vitamin A in the presence of fortified oil, as well as the prevalence of vitamins and minerals in the presence of fortified wheat flour (vitamin B12, folate, iron, and zinc).

In **Table 2**, we calculated the additional intake of vitamins or minerals from fortified food by multiplying the amount of fortified food consumed by the amount of nutrients or minerals added during fortification. We used East African Standards for fortification and accounted for the percentage of losses occurring between production and consumption. Uganda values were used due to limited information on percentage loss in Tanzania. We accounted for a 30% loss at the household level for vitamin A in fortified oil and a 15% loss for vitamin B12 and folate in fortified wheat flour. We calculated the nutrient content of each food item separately using TFCT and UFCT. Then, we multiplied the amount consumed, nutrient content, and USDA nutrient retention factor based on the cooking method. Finally, we divided the product by 100 to obtain nutrient intake in grams. We used a formula to calculate the absolute magnitude of the nutrient gap at Q1, which involved subtracting the Q1 usual nutrient intake from the Abs(H-AR value). To represent the magnitude of the nutrient gap at Q1 as a percentage of the H-AR, we used another formula - [(absolute magnitude nutrient gap at Q1/H-AR value) *100]. For energy, we used the RDA value instead of the H-AR value, which was not available.

We analyzed the responses of 500 respondents interviewed during the first visit to determine the percentage of the study population with inadequate nutrient intakes between TFCT and UFCT. We used descriptive statistical analysis and Pearson’s chi-square test to supplement our findings. A threshold of p-value <0.001 was retained to determine whether the percentage of the study population with inadequate nutrient intakes between TFCT and UFCT differed significantly. We also used a double-sided T-test to assess if the first and third quartiles (Q1 and Q3) of nutrient intakes by TFCT and UFCT showed significant differences, with a corresponding Levene p-value <0.01. Finally, we presented results in both weighted and unweighted values.  We used the Collapse Stata syntax to sum the total values for each nutrient intake. Next, we estimated the usual gram intake of nutrients using the National Cancer Institute (NCI) SAS macro. We used the SAS Studio version 9.4 (SAS Institute Inc. 2013) to analyze the probability of adequate intake of the key nutrients of interest (energy, protein, and 12 micronutrients). However, because the iron requirements distribution is not symmetrical, we used the Simulating Intake of Micronutrients for Policy Learning and Engagement (SIMPLE) SAS macro developed by the University of California, Davis to assess iron intake.

**Results**

We have compiled a comprehensive list of 137 different food items. Out of these, 71 are directly reported as single foods, and the remaining 66 are distinct ingredients extracted from recipes. Among the recipes, 87 involve the use of oil (vegetable oil, sunflower oil, or palm oil), 15 are homemade recipes that use sugar, 9 are industrial recipes that use sugar, and 13 recipes use wheat flour. Additionally, we have recorded 10 different cooking or preparation methods. **Table 3** includes the updated number of recipes and single foods and compares them with the initial list to highlight any discrepancies.

**Table 4** shows the consumption of three different types of food that could be used for large-scale fortification. All women reported consuming oil, with consumption levels in the first and third quartiles being less than 36gm and 37gm, respectively. Consumption levels were similar in both urban and rural areas, with the mean and median consumption being 36.5gm. Our findings indicate that slightly over 20% of women (110 out of 500) consumed food containing wheat flour, with the first quartile value being less than 70.2gm and the third quartile value being less than 209.8gm. More urban women (31%) reported consuming wheat flour than rural women (17%), with urban consumers also reporting significantly higher consumption levels than their rural counterparts in both the first quartile (73.4gm versus 57.9gm) and third quartile (284.5gm versus 142.2gm).

**Table 5** displays the median, Q1, and Q3 for the typical daily nutrient intakes of all women categorized by their place of residence, i.e., urban and rural areas. The survey found that the median energy intake among all the women was 2,169.9 kcal, and there was no difference in the intake between women residing in urban and rural areas (2,173 kcal versus 2,169.2 kcal). Women in urban areas had higher values in Q1 and Q3 than those in rural areas. The median protein intake was 63.5gm, almost the same for urban and rural women. The Q3 intake of usual energy was higher than the expected energy expenditure for women in both urban and rural areas. The Q3 intake was 6.5mg for vitamin E, 17.02mg for iron, and 1.7g for copper. These values were similar for urban and rural women and were below the recommended H-AR thresholds.

**Table 6** presents the percentage of participants in this study who had insufficient intake for each nutrient. The prevalence of inadequacy ranged from 1.9% for protein to 91.2% for zinc. The highest prevalence of inadequate intake was found for zinc (91.2%), vitamin E (89.9%), iron (82.2%), vitamin B12 (80%), and riboflavin (54.8%). About one-third of the women lacked sufficient amounts of energy, folate, and thiamine. Additionally, one-fourth of them were deficient in vitamin A, niacin, vitamin C, and vitamin B6. We calculated both the absolute and the percentage of H-AR magnitude of nutrient gaps in Q1 to illustrate the severity of nutrient inadequacy among the women in our study. Q1's absolute nutrient gap ranged from 0.2 mg for thiamine to 70.1 kcal for energy. The Q1 magnitude of the nutrient gap was highest for vitamin E (50.7% of H-AR, 6.1 mg), vitamin C (42.3% of H-AR, 32.8 mg), and riboflavin (40.9% of H-AR, 0.6 mg). Between 30% and 40% of H-AR were for niacin, vitamin B12, and iron. For vitamin A, the absolute nutrient gap was 159.9μg, with a magnitude gap of 32.6% of H-AR. For vitamin B6, the absolute nutrient gap was 0.3mg, with a magnitude gap of 26.5% of H-AR. For folate, the absolute nutrient gap was 40.5μg, with a magnitude gap of 16.2% of H-AR. Finally, for zinc, the absolute gap was 1mg, with a magnitude gap of 9.8% of H-AR.

**Table 7** compares the median usual nutrient intake using the TFCT and UFCT.  Levene's test was used for each nutrient's median usual consumption. When only non-fortified foods (NFF) or diet is considered, the median usual intake of energy intake, vitamin A, riboflavin, niacin, vitamin B6, vitamin C, and folate showed significant differences between TFCT and UFCT (at a p-value of <0.05). However, when fortified foods are considered, the median usual intake of vitamin A (RAE), vitamin C, and iron showed significant differences between TFCT and UFCT (at a p-value of <0.05). On other nutrients, there were no significant differences in the median usual intake between TFCT and UFCT.

**In Table 8,** the percentage of people who don't get enough vitamin A under three different scenarios: 1) only through their diet, without fortified foods, using the TFCT, 2) through their diet and fortified oil, using the TFCT method, and 3), using the UFCT.  When looking at diet alone, the median vitamin A intake was 772.3μg. As shown in Tables 5 and 6, around 37% of people had an inadequate intake of vitamin A. However, when oil was introduced into their diet using the TFCT, the median vitamin A intake increased to 1848.9μg. When the UFCT was used, the median vitamin A intake increased to 2541.1μg. As a result, the prevalence of inadequate vitamin A intake decreased to 7.4% and 5.6%, respectively, in the last two scenarios. This finding highlights the benefits of consuming oil fortified with vitamin A.

**Table 9** shows the significant reduction in inadequate intake of vitamin B12 and folate due to the fortification of wheat flour with the Tanzania Food and Drugs Authority (TFDA) formula. Before fortification, the median usual intake of folate was 398.2μg. However, after fortification, it increased to 493.5μg, which is a statistically significant increase at p-value <0.01, and the prevalence of inadequate intake decreased from 32.6 to 25.8 percent. Similarly, the median usual intake of vitamin B12 increased from 1.4μg to 2.6μg, and the prevalence of inadequate intake decreased from 78.8 to 0.4 percent, which is also statistically significant at p-value <0.01. For zinc, the median usual intake increased from 9.63mg to 10.5mg, which resulted in a substantial decrease in the prevalence of inadequate zinc intake from 90.6 percent to 27.8 percent. It is important to note that the wheat flour fortification formula in Tanzania does not include thiamine, riboflavin, niacin, and vitamin B6, and the inadequacy of all these micronutrients, although mild, was around 30–40 percent.

**Discussion**

This study highlights a significant prevalence of inadequate micronutrients such as zinc, vitamin E, iron, vitamin B12, and riboflavin among women of reproductive age in the Mbeya Region of Tanzania who are not pregnant or lactating. The insufficient intake of these micronutrients can be attributed to lower consumption of meat, dairy products, fish, poultry, whole grains, and dark green leafy vegetables [24-27]. To address this issue, it is essential to ensure adequate consumption of these foods, which can help alleviate these deficiencies among women of reproductive age in Tanzania. Promoting the year-round availability of vitamin C-rich foods such as oranges, papaya, cabbage, and green leafy vegetables could be of paramount importance as this could enhance the absorption of non-heme iron found in foods such as nuts, beans, or amaranth leaves, which are commonly consumed by women of reproductive age in Mbeya [28].

We gathered data during the dry season, but it's worth noting that seasonal changes could significantly affect the results of a 24-hour recall consumption survey. For instance, the availability of vitamin A-rich fruits or vegetables may fluctuate greatly depending on the season. Some fruits or vegetables may be abundant during specific periods, but scarce or absent during other times. This variability could introduce bias in usual dietary intake, leading to inaccurate nutrient intake [22,23]. However, the NCI usual intake model takes this issue into account and adjusts for days when dietary data might be missing, recognizing that people don't eat the same way every day and that food availability changes with the seasons [22,23].

Our research findings are consistent with several studies conducted in different parts of the world. Goh et al. (2023) study reported that non-pregnant and non-lactating women of reproductive age (NPNL WRA) had insufficient dietary intake of essential micronutrients such as iron, zinc, and vitamin B12 [26]. Similarly, Otunchieva et al. (2022) reported that WRA in low-income countries experienced micronutrient deficiencies [16]. Another study by Sharma et al. (2020) revealed that a large percentage of WRA worldwide had less than the recommended dietary intake of iron [18]. In southeast Nigeria, Onyeji and Sanusi (2022) found low micronutrient intake among WRA, with urban women having slightly higher energy and protein intake than their rural counterparts [15]. This is consistent with findings from another study conducted among WRA in Vietnam by Nguyen et al. (2013) [14].

It is crucial to examine the nutrient gap in the first quarter (Q1) as it is the gap that needs to be filled with micronutrient-delivering interventions. This study emphasizes the significance of multi-faceted interventions to ensure adequate intake of nutrients, especially vitamin E, iron, vitamin B12, riboflavin, and vitamin C. For example, the study shows a relatively low level of zinc inadequacy (9.8 percent of H-AR), indicating that the high prevalence of inadequate zinc intake (91.2 percent) could be significantly reduced through food fortification and improved dietary practices [29].

After analyzing the data from our survey, we observed that people are consuming more vitamin A-rich foods such as carrots, sweet potatoes with orange flesh, and various green leafy vegetables like pumpkin leaves, spinach, and amaranth leaves. Despite the increase in consumption of these foods, vitamin A deficiency remains a concern. Therefore, fortification of vitamin A in food can be a potential solution to prevent its deficiency [29-31].

All the women surveyed reported that they consumed oil and the intake was similar in urban and rural areas. This is because almost every recipe they used contained oil as a basic ingredient in food preparation. Vegetable oil or palm oil was the most commonly used oils, extracted from a variety of foods. The study found that women in rural areas consumed higher amounts of sugar than those in urban areas [32-36]. The sugar intake included all sugars added to foods or drinks, whether industrially manufactured or added in homemade foods, as well as sugars added in fruit juices and fruit juice concentrates.

The study also assessed the consumption of fortified foods, including edible oil and wheat flour. In 2015, the consumption of edible oil among women of reproductive age was 22 g/day, and for wheat flour, it was 162 g/day [7]. However, these numbers were computed using a different methodology than the one used in this study. The study also found a low coverage, use, and intake of wheat flour, although the average quantity of intake in the consumers was higher among urban women.

The study further highlighted that the consumption of fortified foods (oil and wheat flour) led to increased intake of vitamins A and B12 and zinc, and decreased the prevalence of inadequate intake compared to the values of non-fortified foods. As part of the data-cleaning process, the study developed various tools such as a comprehensive list of recipes with their ingredient breakdowns and the harmonized conversion of local measurements to standard units. These tools can potentially improve and refine standardized Tanzania 24-hour recall food intake survey instruments, including multi-pass questionnaires, a food photo book, and a Kobo tablet-based data entry template.

Our study has certain limitations in the collection and analysis of data. Nevertheless, we have taken measures to reduce their impact on our results to the maximum possible extent. Firstly, we did not gather information regarding the specific brands of oil or wheat flour (vehicles for fortification) that the women consumed. Therefore, we utilized the quantities of fortificants required by government regulations (and not the actual level of fortification) in order to conduct a simulation analysis to estimate the potential effect of fortified oil on inadequate intake of vitamin A, and fortified wheat flour on inadequate intake of vitamin B12, folate, iron, and zinc. We also included thiamine, riboflavin, niacin, and vitamin B6 to demonstrate that their inadequacies remain high because fortified wheat flour does not contain these micronutrients. We followed the recommended range of values for micronutrient fortificants (such as vitamin A and others) in our simulation analysis.

Second, we discovered some inaccuracies in the nutrient content values of TFCT and implemented several measures to address them. To correct the caloric content, we used the 4:4:9:2 rule, which involves multiplying the protein, carbohydrate, fat, and total fiber content in grams by 4, 4, 9, and 2 kcal/gram, respectively. We also examined the bioconversion factor of pro-vitamin A carotenoid to retinol in TFCT and UFCT. Finally, we conducted a comparative analysis between TFCT and UFCT to identify potential discrepancies or confirm the validity of intake inadequacy findings. However, we couldn't compare the vitamin E and copper content of TFCT and UFCT because there was no information on these two elements in UFCT.

Third, our findings were based on the consumption data of NPNL WRA in the Mbeya Region only. Therefore, it would be inappropriate to generalize these findings to comment on the dietary patterns, behaviours, and nutrient intakes of the general population or other population sub-groups. We also noticed a specific limitation of zero value or missing data management in the episodic SAS macro for riboflavin and vitamin B12. The episodic model is designed to estimate the usual intake for nutrients or foods that are infrequently consumed, and it assumes that missing data or zero value is either completely random or systematic. To address this limitation, we conducted various scenarios. In our preliminary findings, we followed the NCI guideline’s advice and adjusted zero values to half of the minimum values. We also set the number of bootstrap tests at 100. However, our preliminary results showed that 100 percent of riboflavin values exceeded the H-AR threshold of 1.4, while 100 percent of vitamin B12 values fell below the threshold of 2. Consequently, we opted to retain the zero values, but we doubled the number of bootstrap tests from 100 to 200 and amplified the weight coefficient by a factor of 100. These adjustments resulted in a realistic prevalence of inadequacy intake for the nutrients in this report. However, it is important to approach the interpretation and use of these results with caution, as our data may not strictly adhere to the completely random pattern required for the episodic SAS macro. This deviation could potentially introduce bias and lead to less robust estimates.
**Conclusion**

Our study has shown a highly prevalent of inadequate intake of micronutrients among non-pregnant and non-lactating women of reproductive age in Mbeya region, Tanzania. The findings highlight the need for increased attention towards the intake of vitamin E, iron, and vitamin B12, followed by riboflavin and vitamin C, based on the prevalence of inadequate intake. However, for all cases, the highest nutrient gap is lower than 50 percent H-AR for these micronutrients and lowers than 35 percent for the other vitamins of the B-complex (thiamine, niacin, vitamin B6, and folate). The low inadequacy of zinc intake could be easily rectified due to its very low magnitude nutrient gap. Moreover, our study suggests that the prevalence of inadequate intake of vitamin A can be significantly reduced among women given the usual intake of oil. In this context, oil emerges as a promising candidate for fortification. However, fortified wheat flour would have a limited impact on the nutrient gaps observed due to its limited reach among non-pregnant and non-lactant WRA in Mbeya. Nonetheless, further validation analysis of episodic nutrient consumption is still needed to enhance the reliability and robustness of the usual intake values, particularly for riboflavin and vitamin B12, within the context of Mbeya region, Tanzania.

**Reference**

1. Black RE, Victora CG, Walker SP, Bhutta ZA, Christian P, De Onis M, et al. Maternal and child undernutrition and overweight in low-income and middle-income countries. The lancet. 2013;382:427-51.
2. Harika R, Faber M, Samuel F, Kimiywe J, Mulugeta A, Eilander A. Micronutrient status and dietary intake of iron, vitamin A, iodine, folate and zinc in women of reproductive age and pregnant women in Ethiopia, Kenya, Nigeria and South Africa: a systematic review of data from 2005 to 2015. Nutrients. 2017;9:1096.
3. World Health Organization. The State of Food Security and Nutrition in the World 2021: Transforming food systems for food security, improved nutrition and affordable healthy diets for all. Food & Agriculture Org. 2021.
4. Stadlmayr B, Charrondiere UR, Eisenwagen S, Jamnadass R, Kehlenbeck K. Nutrient composition of selected indigenous fruits from sub‐Saharan Africa. Journal of the Science of Food and Agriculture. 2013;93:2627-36.
5. Mehboob R, Gilani SA, Khalid S, Hassan A, Alwazzan A. Maternal mortality ratio in low income developing countries. Global Women’s Health. 2021;10.
6. Allen LH, Carriquiry AL, Murphy SP. Perspective: proposed harmonized nutrient reference values for populations. Advances in Nutrition. 2020;11:469-83.
7. National Bureau of Statistics. Tanzania Demographic and Health Survey and Malaria Indicator Survey (TDHS-MIS) 2015–16.
8. National Bureau of Statistics. Tanzania Demographic and Health Survey and Malaria Indicator Survey (TDHS-MIS) 2010
9. World Health Organization. Anaemia Policy Brief. Global Nutrition Targets 2025, 2(WHO/NMH/NHD/14.4). Geneva: World Health Organization.2014; pp. 8
10. Sunguya BF, Ge Y, Mlunde LB, Mpembeni R, Leyna GH, Poudel KC, et al. Targeted and population-wide interventions are needed to address the persistent burden of anemia among women of reproductive age in Tanzania. International journal of environmental research and public health. 2022;19:8401.
11. Panth M, Shatrugna V, Yasodhara P, Sivakumar B. Effect of vitamin A supplementation on haemoglobin and vitamin A levels during pregnancy. British Journal of Nutrition. 1990; 64:351-8.
12. The United Republic of Tanzania (URT), Ministry of Finance and Planning, Tanzania National Bureau of Statistics and President’s Office - Finance and Planning, Office of the Chief Government Statistician, Zanzibar. The 2022 Population and Housing Census: Administrative Units Population Distribution Report; Tanzania, 2022.
13. Osei-Kwasi H, Mohindra A, Booth A, Laar A, Wanjohi M, Graham F, et al. Factors influencing dietary behaviours in urban food environments in Africa: a systematic mapping review. Public Health Nutrition. 2020;23:2584-601.
14. Nguyen PHG, Strizich A, Lowe H, Nguyen H, Pham TV, Truong S, et al. “Food Consumption Patterns and Associated Factors among Vietnamese Women of Reproductive Age.” Nutrition Journal. 2013;12:126.
15. Onyeji GN and Sanusi RA. “Nutrient Adequacy of Diets of Women of Childbearing Age in South-East Nigeria. Journal of Nutrition and Internal Medicine. 2022;24:e2022085.
16. Otunchieva A, Smanalieva J, and Ploeger A. “Dietary Quality of Women of Reproductive Age in Low-Income Settings: A Cross-Sectional Study in Kyrgyzstan.” Nutrients. 2022;14:289.
17. Ryan-Harshman M. and Aldoori W. “New Dietary Reference Intakes for Macronutrients and Fibre.” Canadian Family Physician. 2006;52:177–9.
18. Sharma S, Faiyaz A, Rajesh KS, and Sunil M. 2020. “Dietary Intake across Reproductive Life Stages of Women in India: A Cross-Sectional Survey from 4 Districts of India.” Journal of Nutrition and Metabolism. 2020:9549214. doi.org/10.1155/2020/9549214.
19. Mbwana HA, Kinabo J, Lambert C, Biesalski HK. Determinants of household dietary practices in rural Tanzania: Implications for nutrition interventions. Cogent Food & Agriculture. 2016;2:1224046.
20. Lwanga SK, Lemeshow S, World Health Organization. Sample size determination in health studies: a practical manual. World Health Organization; 1991.
21. Vossenaar M, Lubowa A, Hotz C, Deitchler M, Moursi M, Arimond M, et al. Considerations for the selection of portion size estimation methods for use in quantitative 24-hour dietary recall surveys in low-and middle-income countries. Washington, DC: Intake–Center for Dietary Assessment/FHI Solutions; 2020.
22. Deitchler M, Arimond M, Carriquiry A, Hotz C, Tooze JA. Planning and Design Considerations for Quantitative 24-Hour Recall Dietary Surveys in Low- and Middle-Income Countries. Washington, DC: Intake—Center for Dietary Assessment/FHI Solutions; 2020.
23. De Keyzer W, Huybrechts I, De Vriendt V, Vandevijvere S, Slimani N, Van Oyen H, De Henauw S. Repeated 24-hour recalls versus dietary records for estimating nutrient intakes in a national food consumption survey. Food & nutrition research. 2011;55:7307..
24. Tontisirin K, Nantel G, Bhattacharjee L. Food-based strategies to meet the challenges of micronutrient malnutrition in the developing world. Proceedings of the Nutrition Society. 2002;61:243-50.
25. Bost M, Sabine H, Marion O, Esther K, Jean-François H, and Irène M. “Dietary Copper and Human Health: Current Evidence and Unresolved Issues.” Journal of Trace Elements in Medicine and Biology. 2016;35:107–115.
26. Goh YE, Manger MS, Duggal M, Das R, Saklani S, Agarwal S, et al. “Women in Selected Communities of Punjab, India Have a High Prevalence of Iron, Zinc, Vitamin B12, and Folate Deficiencies: Implications for a Multiply-Fortified Salt Intervention.” Nutrients. 2023 ;15: 3024.
27. Harvey P, Zo R and Omar D. The 2008 Uganda Food Consumption Survey: Determining the Dietary Patterns of Ugandan Women and Children. Washington, DC: A2Z: The USAID Micronutrient and Child Blindness Project/AED; 2010.
28. Abdallah F, John SE, Hancy A, Paulo HA, Sanga A, Noor R, et al. Prevalence and factors associated with anaemia among pregnant women attending reproductive and child health clinics in Mbeya region, Tanzania. PLOS Global Public Health. 2022;2:e0000280.
29. Global Alliance for Improved Nutrition (GAIN),  Centers for Disease Control and Prevention (CDC), Africa Academy of Public Health (AAPH), Ifakara Health Institute, and National Bureau of Statistics (NBS). 2015. Fortification Assessment Coverage Tool (FACT) Survey in Tanzania; 2015. URL: <https://www.nbs.go.tz/nbs/takwimu/references/FACTSURVEY2015/FACTReport2015.pdf>
30. Dary O, Mora JO. Food fortification to reduce vitamin A deficiency: International Vitamin A Consultative Group recommendations. The Journal of nutrition. 2002;132:2927S-33S.
31. Chakravarty I. Food-based strategies to control vitamin A deficiency. Food and nutrition Bulletin. 2000;21:135-43.
32. Lassi, ZS, Salam RA, Das JK, Wazny K and Bhutta ZA. “An Unfinished Agenda on Adolescent Health: Opportunities for Interventions.” Seminar in Perinatology. 2015; 39:353–360.
33. Ministry of Health of the United Republic of Tanzania (MOH). Tanzania Mainland Food-Based Dietary Guidelines for a Healthy Population: Technical Recommendations. Ministry of Health: Dodoma, Tanzania; 2023.
34. U.S. Department of Agriculture (USDA) “Food and Nutrition Information Center.”URL: <https://www.nal.usda.gov/programs/fnic> (Accessed November, 2023)
35. World Health Organization (WHO). Guideline: Sugars Intake for Adults and Children*.* Geneva: World Health Organization; 2015.
36. World Health Organization (WHO). “Healthy Diets”; 2020.URL:<https://www.who.int/news-room/fact-sheets/detail/healthy-diet> (Accessed November 7, 2023).

**Acknowledgement**

We extend our sincere appreciation to the non-pregnant, non-lactating women, aged 15 to 49 years, residing in Chunya DC, Mbeya DC, Mbeya City, Mbarali DC, Kyela DC, Rungwe DC, and Busokelo DC of Mbeya Region, along with all those who collaborated with us during this project. We express our gratitude to USAID and USAID Advancing Nutrition, for their invaluable guidance and technical support in the development and implementation of this study.

**Declarations:**

**Institutional Review Board Statement**

This study obtained ethical permit from the National Health Research Ethics Review Committee (NatHREC) at National Institute for Medical Research with reference no. NIMR/HQ/R.8a/Vol.IX/3964.

**Informed Consent Statement**

All study participants were asked to provide written informed consent after being read a document emphasising the voluntary nature of the survey. Furthermore, written informed consent was obtained from the parent/guardian of each participant under 18 years of age. Anonymity was maintained throughout the study and data sets will be destroyed when the project is concluded.

**Data Availability Statement**

Available here, URL: https://www.advancingnutrition.org/sites/default/files/2023-09/usaid_an_intake_survey_tz_2023.pdf

**Competing interests**

The authors declare that they have no competing interests.

**Funding**

The U. S. Agency for International Development provided financial support for this article through its flagship multi-sectoral nutrition project, USAID Advancing Nutrition. It was prepared under the terms of contract 7200AA18C00070 awarded to JSI Research & Training Institute, Inc. with a subgrant award to the Tanzania Food and Nutrition Centre (TFNC). The contents are the responsibility of the authors and do not necessarily reflect the views of USAID or the U.S. Government.

**Authors’ contributions**

**Conceptualization:** Nyamizi J. Ngassa, Ray M. Masumo, Germana H. Leyna. **Formal analysis:** Nyamizi J. Ngassa,, Ray M. Masumo, Adam Hancy, Esther J. Kabula, Erick Killel, Jackline Nusurupia, Adeline Munuo, Hoyce Mshida, Rose Mpembeni and Germana H. Leyna. **Investigation:** Nyamizi J. Ngassa,, Ray M. Masumo, Adam Hancy, Esther J. Kabula, Erick Killel, Jackline Nusurupia, Adeline Munuo, Hoyce Mshida, Rose Mpembeni, Elizabeth lyimo, Rose V. Msaki, Theresia Jumbe and Germana H. Leyna. **Methodology:** Nyamizi J. Ngassa,, Ray M. Masumo, Adam Hancy, Esther J. Kabula, Erick Killel, Rose Mpembeni, Theresia Jumbe and Germana H. Leyna **Project administration:** Germana Leyna, Theresia Jumbe. **Supervision:** Ray M. Masumo, Rose Mpembeni, Germana H. Leyna. **Writing – original draft:** Nyamizi J. Ngassa,, Ray M. Masumo, Adam Hancy, Esther J. Kabula, Erick Killel, Jackline Nusurupia, Adeline Munuo, Hoyce Mshida, Rose Mpembeni, Elizabeth lyimo, Rose V. Msaki, Theresia Jumbe and Germana H. Leyna. All authors have read and agreed to the published version of the manuscript.

**Consent for publication**

Not applicable

**Supporting information**

S1 Strobe checklist

**LIST OF TABLES**

**Table 1: Recommended Daily Allowance (RDA) and Harmonized Average Requirement Values by Age Group**

| **Nutrients** | **Adolescents Aged 15–19 Years** | **Women Aged 20–49 Years** |
| --- | --- | --- |
| **Macronutrients (RDA) *** |  |  |
| Energy (kilocalorie [kcal]) | 2200 | 2100 |
| Protein (g) (H-AR) | (0.8 X 60 kilogram (kg)** = 48 g/day) | (0.8 X 70 kg*** = 56 g/day) |
| **Micronutrients (H-AR Values)** | | |
| Vitamin A retinol activity equivalent (RAE) (microgram [μg]) | 490 | 490 |
| Vitamin B1—Thiamine (milligram [mg]) | 0.9 | 0.9 |
| Vitamin B2—Riboflavin (mg) | 1.4 | 1.3 |
| Vitamin B3—Niacin (mg) | 11 | 11 |
| Vitamin B6 (mg) | 1.3 | 1.3 |
| Vitamin B12 (μg) | 2 | 2 |
| Vitamin C (mg) | 75 | 80 |
| Vitamin E (mg) | 12 | 12 |
| Copper (μg) | 685 | 700 |
| Folate dietary folate equivalent (μg) | 250 | 250 |
| Iron (mg) (low absorption—5 percent) | 22.4 | 22.4 |
| Zinc (mg) (unrefined diet, 1200 mg phytate/day in the diet) | 10.2 | 10.2 |

* Ryan-Harshman and Aldoori 2006

** The average weight of women in Tanzania aged 15 to 19 years old (MoH 2023), *** the average weight of women in Tanzania aged 20–49 years old (MoH 2023)

**Table 2: Amounts of Fortified Vitamins and Minerals Added at the Factory and Estimated Percentage Losses at the Household Level**

| **Nutrients** | **Added at the Factory**  **(mg/100 g)** | **Estimated Losses at the Household Level** |
| --- | --- | --- |
| **Vegetable Oil** |  |  |
| Vitamin A | 2.5 | 30% |
| **Wheat Flour** |  |  |
| Vitamin B1—Thiamine | 0.86 | 0% |
| Vitamin B2—Riboflavin | 0.4945 | 0% |
| Vitamin B3—Niacin | 4.945 | 0% |
| Vitamin B6—Pyridoxine | 0.559 | 0% |
| Vitamin B12— Cyanocobalamin | 0.00129 | 15% |
| Folate—Folic acid | 0.258 | 15% |
| Iron—Ferrous fumarate | 3.44 | 0% |
| Zinc—Zinc oxide | 4 | 0% |

**Source:** As specified in the 2008 Uganda Food Consumption Survey (Harvey, Rambelson, and Dary 2010)

**Table 3: Numbers of Recipes/Single Foods by List Type**

| **Recipes/Food Items** | **Number in the Initial List** | **Number in the Updated List** |
| --- | --- | --- |
| **Fortification Vehicles and Sugar** |  |  |
| Recipes containing **oil** | 49 | 87 |
| Recipes containing **wheat flour** | 13 | 13 |
| Homemade recipes containing **sugar** | 15 | 15 |
| Industrially made foods **^a^** containing **sugar** | 5 | 9 |
|  |  |  |
| **General Recipes/Single Foods** |  |  |
| Total recipes | 62 | 113* |
| Total reported single foods | 70 | 71 |
| Total single foods extracted from recipes | 24 | 66 |
| **Total single foods** | **94** | **137** |

**^a^** biscuit, bread—brown, bread—roll, bread—white, candy, chocolate, ice cream, and soda

* The number of recipes increased when we reconstructed the recipes based on the actual ingredients reported by women.

**Table 4: The Consumption of Food Considered by Fortification Vehicle and Sugar (Homemade and Industrially Made) among the NPNL WRA of Each Food**

| **Food Item** | **Residence** | | | | | | | | **Total** | | | |
| --- | --- | --- | --- | --- | --- | --- | --- | --- | --- | --- | --- | --- |
|  | **Rural (N = 319)** | | | | **Urban (N = 181)** | | | | **(N = 500)** | | | |
|  | **n (%)** | **Mean (g) Median (g)** | **Q1 (g)** | **Q3 (g)** | **n (%)** | **Mean (g)**  **Median (g)** | **Q1 (g)** | **Q3 (g)** | **n (%)** | **Mean (g) Median (g)** | **Q1**  **(g)** | **Q3**  **(g)** |
| Oil^1^ | 319 (100) | 36.50 | 35.99 | 37.02 | 181(100) | 36.54 | 36.12 | 37.08 | 500 (100) | 36.51 | 36.01 | 37.04 |
|  |  | 36.45 |  |  |  | 36.50 |  |  |  | 36.47 |  |  |
| Wheat flour^2^ | 53 (17) | 131.29 | 57.89 | 142.23 | 57(31) | 234.07 | 73.68 | 284.45 | 110 (25) | 184.55 | 70.23 | 209.8 |
|  |  | 86.84 |  |  |  | **142.23***** |  |  |  | 110.53 |  |  |
| Sugar— Home^3^ | 319 (100) | 63.59 | 36.91 | 74.39 | 181 (100) | 60.55 | 38.06 | 69.06 | 500 (100) | 62.49 | 37.39 | 72.7 |
|  |  | 51.54 |  |  |  | 52.42 |  |  |  | 51.66 |  |  |
| Sugar— Industrial^4^ | 28 (9) | 27.27  19.89 | 18.30 | 37.55 | 29 (16) | **21.80***  **18.30**** | 18.30 | 24.76 | 57 (11) | 24.49  19.10 | 18.30 | 33.0 |

* *p*-value <0.05 for Levene test centred at the mean (i.e., W0), ** *p*-value <0.05 for Levene test at the median (i.e., W50), *** *p*-value <0.01 for Levene test at the median (i.e., W50)

**^1^ Oil** includes vegetable oil and palm oil, extracted from a long list of foods prepared with oil. The analysis considered usual intake based on the overall trend.

**^2^ Wheat flour** includes wheat flour and food made from wheat flour. The analysis considered the observed grams consumed by each reported respondent.

**^3^ Sugar—home** includes sugar extracted from a long list of foods, which are non-industrially prepared with sugar. The analysis considered usual intake based on the overall trend.

**^4^ Sugar—industrial** includes sugar extracted from biscuit, bread—brown, bread—rolls, bread—white, candy, chocolate, ice cream, and soda. The analysis considered the observed grams consumed by each reporting respondent.

**Table 5: Usual Macronutrient and Micronutrient Intake by Place of Residence**

| **Nutrient (Measurement)*** | **H-AR/RDA** | | **Residence** | | | | | | **Total** | | |
| --- | --- | --- | --- | --- | --- | --- | --- | --- | --- | --- | --- |
|  | **Adolescent 15–19 Years Old** | **Women 20–49 Years Old** | **Rural (N = 319)** | | | **Urban (N = 181)** | | | **(N = 500)** | | |
|  |  |  | **Mean [Median]** | **Q1** | **Q3** | **Mean [Median]** | **Q1** | **Q3** | **Mean [Median]** | **Q1** | **Q3** |
| Energy (kcal) | 2200 | 2100 | 2171.8  [2169.2] | 2069.9 | 2270.2 | 2176.7  [2173.5] | 2091.2 | 2255.1 | 2173.6  [2169.9] | 2079.9 | 2263.5 |
| Protein (g) | 48 | 56 | 63.6  [63.5] | 59.8 | 67.2 | 63.8  [63.7] | 60.6 | 66.7 | 63.7  [63.5] | 60.2 | 67.0 |
| Vitamin A (μg [RAE]) | 490 | 490 | 1128.6  [733.4] | 324.3 | 1380.3 | 1147.1  [804.2] | 343.1 | 1505.5 | 1135.3  [772.3] | 330.1 | 1430.3 |
| Thiamine (mg) | 0.9 | 0.9 | 1.1  [1.3] | 0.7 | 1.4 | 1.1  [1.3] | 0.7 | 1.4 | 1.1  [1.3] | 0.7 | 1.4 |
| Riboflavin (mg) | 1.4 | 1.3 | 1.7  [1.2] | 0.8 | 2.7 | 1.7  [1.2] | 0.8 | 2.7 | 1.7  [1.2] | 0.8 | 2.7 |
| Niacin (mg) | 11 | 11 | 10.4  [12.4] | 7.3 | 13.6 | 10.4  [12.5] | 6.7 | 13.6 | 10.4  [12.4] | 7.3 | 13.6 |
| Vitamin B6 (mg) | 1.3 | 1.3 | 1.2  [1.5] | 1.0 | 1.6 | 1.2  [1.5] | 1.0 | 1.6 | 1.2  [1.5] | 1.0 | 1.6 |
| Vitamin B12 (μg) | 2 | 2 | 2.1  [1.4] | 1.3 | 1.5 | 2.5  [1.4] | 1.3 | 2.2 | 2.2  [1.4] | 1.3 | 1.5 |
| Vitamin C (mg) | 75 | 80 | 78.7  [102.8] | 46.6 | 111.1 | 78.7  [105.2] | 42.5 | 110.9 | 78.7  [103.9] | 44.7 | 111.0 |
| Vitamin E (mg) | 12 | 12 | 7.7  [6.2] | 5.9 | 6.5 | 7.8  [6.2] | 5.9 | 6.5 | 7.7  [6.2] | 5.9 | 6.5 |
| Copper (g) | 0.685 | 0.7 | 1.7  [1.7] | 1.6 | 1.7 | 1.7  [1.6] | 1.6 | 1.7 | 1.7  [1.7] | 1.6 | 1.7 |
| Folate (μg) | 250 | 250 | 328.5  [397.8] | 209.7 | 419.6 | 328.7  [401.6] | 208.2 | 422.1 | 328.6  [398.2] | 209.6 | 421.0 |
| Iron (mg) | 22.4 | 22.4 | 20.5  [16.0] | 15.4 | 17.2 | 21.0  [16.1] | 15.5 | 16.9 | 20.7  [16.1] | 15.4 | 17.0 |
| Zinc (mg) | 10.2 | 10.2 | 9.2  [9.6] | 9.2 | 9.9 | 9.2  [9.7] | 9.2 | 10.0 | 9.2  [9.6] | 9.2 | 10.0 |

* We used the TFCT to estimate the nutrient intake and did not consider the effect of fortified food.

**Table 6: Usual Nutrient Intake, Prevalence of Intake below the H-AR, and Magnitude of Nutrient Gap at Q1 (N=500, Subsample n=120 for Percent below H-AR Analysis)**

| **Nutrient* (Measurement)** | **Median** | **Q1** | **Q3** | **Prevalence of Inadequate Intake (percent below H-AR)** | | **Absolute Magnitude of Nutrient Gap at Q1^±^** | **Magnitude of Nutrient Gap at Q1 (% H-AR) ^ǂ^** |
| --- | --- | --- | --- | --- | --- | --- | --- |
|  |  |  |  | **Unweighted** | **Weighted** |  |  |
| Energy (kcal) | 2169.88 | 2079.90 | 2263.54 | 34.4 | 33.7 | 70.1 | 3.3 |
| Protein (g) | 63.52 | 60.21 | 66.99 | 2.6 | 1.9 | - | - |
| Vitamin A (μg [RAE]) | 772.26 | 330.12 | 1430.32 | 37.0 | 39.8 | 159.9 | 32.6 |
| Thiamine (mg) | 1.23 | 0.70 | 1.41 | 34.8 | 35.4 | 0.2 | 22.8 |
| Riboflavin (mg) | 1.23 | 0.79 | 2.70 | 53.0 | 54.8 | 0.6 | 41.9 |
| Niacin (mg) | 12.48 | 7.26 | 13.59 | 44.4 | 45.1 | 3.7 | 34.0 |
| Vitamin B6 (mg) | 1.47 | 0.96 | 1.59 | 45.2 | 47.1 | 0.3 | 26.5 |
| Vitamin B12 (μg) | 1.37 | 1.29 | 1.53 | 78.8 | 80.0 | 0.7 | 35.5 |
| Vitamin C (mg) | 103.93 | 44.72 | 111.00 | 45.6 | 46.5 | 32.8 | 42.3 |
| Vitamin E (mg) | 6.18 | 5.91 | 6.51 | 89.0 | 89.9 | 6.1 | 50.7 |
| Copper (g) | 1.65 | 1.58 | 1.73 | 0.0 | 0.0 | - | - |
| Folate (μg) | 398.18 | 209.55 | 420.99 | 32.6 | 34.7 | 40.5 | 16.2 |
| Iron (mg) | 16.05 | 15.39 | 17.02 | 80.0 | 82.2 | 7.0 | 31.3 |
| Zinc (mg) | 9.63 | 9.21 | 9.96 | 90.6 | 91.2 | 1.0 | 9.8 |

*We used the TFCT to estimate the nutrient intake and did **not** consider the effect of fortified food

**^±^** Calculated as H-AR value—Absolute nutrient intake at Q1 (in absolute value). We used the RDA value for energy.

^ǂ^ Calculated as absolute nutrient gap/H-AR or RDA (in percentage)

**Table 7: Comparison of Nutrient Intake from Reported Food Non-Fortified and Those Considered Fortified According to the Tanzania and Uganda Food Composition Tables**

| **Nutrient (Measurement)** | **Non-Fortified Foods (N = 500)** | | | | **Fortified Foods (N = 500)** | | | |
| --- | --- | --- | --- | --- | --- | --- | --- | --- |
|  | **TFCT** | | **UFCT** | | **TFCT** | | **UFCT** | |
|  | **Median (g) + IQR** | | **Median (g) + IQR** | | **Median (g) + IQR** | | **Median (g) + IQR** | |
| Energy (kcal) | 2169.88 | 183.98 | **2238.55*** | 193.22 | 2260.96 | 243.11 | 2265.76 | 232.12 |
| Protein (g) | 63.52 | 6.79 | 63.90 | 7.09 | 66.26 | 7.76 | 66.28 | 7.83 |
| Vitamin A (μg [RAE]) | 772.26 | 1102.1 | **1499.78^a^** | 138.55 | 1848.91 | 155.70 | **2541.06*** | 1871.1 |
| Thiamine (mg) | 1.23 | 0.72 | 1.14 | 0.64 | 1.25 | 0.66 | 1.23 | 0.75 |
| Riboflavin (mg) | 1.23 | 1.91 | **1.08^a^** | 0.07 | 1.23 | 1.91 | 1.16 | 0.07 |
| Niacin (mg) | 12.48 | 6.35 | **13.09^a^** | 6.73 | 12.68 | 6.96 | 12.80 | 6.90 |
| Vitamin B6 (mg) | 1.47 | 0.64 | **1.90^a^** | 0.95 | 1.49 | 0.98 | 1.54 | 0.91 |
| Vitamin B12 (μg) | 1.37 | 0.24 | 1.73 | 0.32 | 2.63 | 0.34 | 2.63 | 0.34 |
| Vitamin C (mg) | 103.93 | 66.37 | **78.32^a^** | 56.94 | 90.40 | 60.20 | **78.32*** | 61.98 |
| Vitamin E (mg) ^†^ | 6.18 | 0.60 | ---- | ---- | 6.18 | 0.60 | ---- | ---- |
| Copper (g) ^†^ | 1.65 | 0.15 | ---- | ---- | 1.65 | 0.15 | ---- | ---- |
| Folate (μg) | 398.18 | 211.60 | **411.79^a^** | 214.96 | 493.46 | 288.53 | 493.81 | 287.40 |
| Iron (mg) | 16.05 | 1.64 | 16.58 | 1.96 | 17.55 | 2.70 | **19.82*** | 4.30 |
| Zinc (mg) | 9.63 | 0.76 | 9.68 | 1.12 | 9.64 | 0.777 | 9.69 | 1.13 |

^†^ Vitamin E and copper were not available in the Uganda FCT

^a^ *p*-value <0.05 non-fortified TFCT vs. UFCT; and ^b^ *p*-value <0.05 fortified TFCT vs. UFCT. The study uses *p*-values computed from the Levene’s test using the median.

**Table 8: Usual Intake and Predicted Prevalence of Inadequate Vitamin A Intake in the Presence of Fortification**

| **Predicted Scenario** | **Vitamin A Intake (N = 500)** | |
| --- | --- | --- |
|  | **Median (g)** | **Inadequacy (%)** |
| Diet alone—NFF TFCT | **772.26^a,b^** | **37%^a,b^** |
| Diet plus fortified oil—FF TFCT | 1848.91 | 7.4% |
| Diet plus fortified oil—FF UFCT | 2541.07 | 5.6% |

^a^ *p*-value< 0.001 non-fortified TFCT vs. fortified TFCT, ^b^ *p*-value< 0.001 non-fortified TFCT vs. fortified UFCT

**Table 9: Usual Intake and Predicted Prevalence of Inadequate Micronutrient Intake in the Presence of Fortification**

| **Nutrient Added** | **NFF TFCT (N = 500)** | | **FF TFCT (N = 500)** | |
| --- | --- | --- | --- | --- |
|  | **Median** | **Inadequacy (%)** | **Median** | **Inadequacy (%)** |
| Thiamine (mg) | 1.23 | 34.8% | 1.25 | 34.8% |
| Riboflavin (mg) | 1.23 | 53.0% | 1.23 | 53.0% |
| Niacin (mg) | 12.48 | 44.4% | 12.68 | 44.4% |
| Vitamin B6 (mg) | 1.47 | 45.2% | 1.49 | 45.2% |
| Vitamin B12 (μg) | 1.37 | 78.8% | **2.63^a^** | 0.4% **^a^** |
| Folate (μg) | 398.18 | 32.6% | **493.46^a^** | 25.8% |
| Iron (mg) | 16.05 | 80.0% | 17.55 | 76.2% |
| Zinc (mg) | 9.63 | 90.6% | 10.57^a^ | 27.8%^a^ |

**^a^** *p*-value <0.001, NFF TFCT vs. FF TFCT
