# Supplementary material for: Food and nutrient intake among non-pregnant, non-lactating women of reproductive age of Mbeya in Tanzania: A repeated 24-hour dietary recall
Source: PLOS Glob Public Health. 2024 Dec 5;4(12):e0004010. doi: 10.1371/journal.pgph.0004010 (PMC11620714; doi:10.1371/journal.pgph.0004010)
Supplement: Attachment — Submitted filename: A rebuttal letter_22_09_2024.docx [file pgph.0004010.s003.docx]

Dear Steve Zimmerman, PhD

Thank you for affording us the opportunity to submit a revised draft of our manuscript entitled “Food and nutrient intake among non-pregnant, non-lactating women of reproductive age in the Mbeya region of Tanzania: A repeated 24-hour dietary recall.”

We extend our sincere appreciation for the time and dedication you and the reviewers have invested in providing valuable feedback on our manuscript.

Herewith is a detailed point-by-point response addressing the reviewers’ comments and concerns.

**Journal Requirements**

**Comment 1:** You indicated that you had ethical approval for your study. In your Methods section, please ensure you have also stated whether you obtained consent from parents or guardians of the minors included in the study or whether the research ethics committee or IRB specifically waived the need for their consent.
**Response**: Thank you for bringing this to our attention. We have revised the method section to explain how consent was obtained from parents or guardians of minors (Page 4; Line 108-109).

**Review Comments to the Author**

**Reviewer #1**

**Comment 1**: The title is inappropriate because the data come from people in only one region of Tanzania and are not nationally representative.
**Response:** Thank you very much for your input. We have agreed with your suggestion and have rewritten the title as appropriate (Page 1; Line 2).

**Comment 2:** The Sample size and sampling procedures and Data Collection sections need to be written more clearly.
How was the sample size of the survey determined? Is it representative?
Only 100 people in total participated in the 24-hour dietary recall survey? 5 people in each EA (15 in total) had a second review?
Were the 24-hour dietary recall conducted on weekdays or weekends, and were the survey days the same in the 3 EAs? Because these may lead to differences in residents' food choices.

**Response:** Thank you for your input. We have revised the sample size and sampling procedures, and also data collection sections accordingly (Page 5-6; Line 117-124 & Line 136-150).

**Comment 3**: Data cleaning and preparation section.
Am I to understand that you investigated the proportion of each food intake and calculated the intake at a later stage? Were specific intakes investigated? If not, how do you avoid the error caused by simply using the reference chart? After all, the actual size is not the same as the printed size.
Were the survey data directly weighed/ recorded by the enumerators in the households or self-reported by the respondents?

**Response:** We acknowledge this as a limitation of our study and have made efforts to mitigate this by providing comprehensive training to data collectors on the utilization of the reference chart (Page 5-6; Line 136-150).

**Comment 4**: Data Analysis section.
I feel that Method part is too long compared to the main text, especially the Data Analysis section. The researcher spends a lot of space describing the recipes and food components tables used in the survey, as well as the treatment of nutrient content. It is recommended to simplify.

**Response:** We have made the edits to the data analysis section as per your suggestions (Page 7-8; Line 179-184).

**Comment 5**: Minor comments

***Minor comment 1*:** Line 43; change “15-49”to “15-49 years”

***Response*:** We have made the necessary edits (Page 3; Line 81).

***Minor comment 2*:** Line 88; delete “(WRA)”

***Response***: We have deleted ‘WRA’. (Page 4; Line 88).

***Minor comment 3***: Line 93-95; “Due to the limited coverage and longer periodicity……diets of targeted populations”. This sentence feels abrupt. I do not understand the function of this sentence and how it connects to the previous and the following sentences.

***Response:*** We appreciate your comment regarding this matter. The confusing sentences have been omitted to enhance the clarity. Thank you for bringing this to our attention.

***Minor comment 4***: Lines 143-144 “The survey was translated from English to Swahili and back translated for accuracy by different translators.” Do different translations lead to different understandings of the problem? Is it standardized?

***Response*:** We have made the edits to the data collection section as per your suggestions (Page 6; Line 142-150).

***Minor comment 5***: Food matching, data quality control and validation section. The use of multiple food composition tables to supplement the sample pool was designed to be thoughtful and comprehensive. This approach ensured that the study covered a wide range of nutrients and food groups, which not only increased the representativeness and accuracy of the results, but also demonstrated methodological forethought and attention to detail on the part of the researchers. However, this design may require consideration of data consistency. How can consistency in nutrient measurement methods and units be ensured for data extracted from different sources of food composition tables? Please add.

***Response*:** We appreciate your comment regarding this matter and rephrased the sentences as per your suggestions (Page 7; Line 168-173).

***Minor comment 6*:** Line 208; the P should be italicized P. Please also check the whole text.

***Respons*e:** We have made correction through the manuscript as per your suggestions.

***Minor comment 7*:** Why did the authors identify nutrient deficiencies and compare differences in results based on two different food composition tables (TFCT and UFCT)? What was the purpose? I did not see this explored in the discussion section

***Response*:** We agree with your comment and have added sentences to improve clarity (Page 7; Line 168-173).

***Minor comment 8*:** Some of the writing is a bit wordy. Be more concise and direct, especially by reducing the use of the first person narration. We employed, we cross-checked, We used, We extracted……

***Response*:** We appreciate your comment on this issue. We have revised our sentences to improve clarity. Thank you for bringing this to our attention.

***Minor comment 9*:** After revising the above points, please show the STROBE guidelines for a cross-sectional study.

***Response*:** The STROBE guidelines have also been revised (Appendix: S1 Strobe checklist).

**Reviewer #2
Comment 1**: The participants were from the seven districts of the Mbeya region of Tanzania. It may not be representative of the entire Tanzania. Thus, it is better to mention the Mbeya region in the title. You can revise the title to “Food and nutrient intake among non-pregnant, non-lactating women of reproductive age of Mbeya region in Tanzania: A repeated 24-hour dietary recall”.

**Response:** Thank you very much for your input. We have agreed with your suggestion and have rewritten the title as appropriate (Page 1; Line 2).

**Comment 2**: The background, rationale, and objective of the study are missing in the abstract. It is recommended to include these points in the abstract.

**Response:** We appreciate your comment regarding this matter and added background, rationale, and objective of the study as per your suggestions (Page 2; Line 30-51).

**Comment 3**: Line #40, please add the full form with the abbreviation when it appears first and then use the abbreviation after forward. Follow this throughout the manuscript where it is applicable.

**Response:** We appreciate your comment on this issue. We have made edits throughout the manuscript where is applicable.

.

**Comment 4**: Lines #125-128, 7 districts, 3 EA from each district, 22 HH from each EA comes to 7*3*22 = 462. But the total number of samples is 500. So there remains confusion.

**Response:** Thank you very much for pointing this out, we have made a small correction, the actual number of HH was 24 (Page 5; Line 127-128).

**Comment 5**: Lines #126-127, the authors mentioned that they used probability proportional to size (PPS). At the same time, they mentioned that an equal number of EA and HH were considered for all. More clarification is needed here.

**Response:** We appreciate your comment on this issue. We have revised our sentences to improve clarity. Thank you for bringing this to our attention.

**Comment 6**: Lines #130-131, please mention how one woman was selected for participation when you found more than one NPNL WRA in the same household.

**Response:** A single NPNL WRA was purposely selected for the study (Page 5; Line 130-131).

**Comment 7:** Lines #131-132, it is not clear whether you obtained data on dietary intake for 100 participants or 500 participants.
**Response:** Thank you very much for your input. We have agreed with your suggestion and have rewritten the sentences for clarity (Page 5; Line 131-134).

**Comment 8**: Lines #132-134, how it is possible to address the effect of seasonality in dietary intake just visiting the household one week after the first visit.

**Response**: We appreciate your comment on this issue. We have revised our sentences to improve clarity. Thank you for bringing this to our attention.

**Comment 9**: Did you conduct any pretesting? If you did so, please give a few points on the location, how many participants, non-response rate, etc.
**Response**: We have made the edits to the data collection section as per your suggestions (Page 5-6; Line 135-150).

**Comment 10**: Lines #172-175, didn't you collect the information regarding the brand of edible oils they used in cooking or the brand of wheat they consumed? Then you can easily find whether they used a fortified one or a non-fortified one. Thus, you can easily avoid the underestimation or overestimation of nutrient intake.

**Response:** Information regarding the brand of edible oils was not collected, as mentioned in the study's limitations (Page 14-15; Line 341-351).

**Comment 11**: It is not clear whether you collected individual dietary recall data or intake data at the household level. Please specify it.

**Response**: We gathered information on what each NPNL WRA in the household ate.

**Comment 12**: Phytate affects the bioavailability of different micronutrients. Please consider this in your findings or at least discuss it as study limitations.

**Response:** Thank you very much and we have added the Phytate affects the bioavailability of different micronutrients into study limitations (Page 14; Line 341-342).

**Comment 13**: The title of the study indicates the food and nutrient intake of the study participants. But no details regarding different food group intake are available in the result section. You included nutrient intake information in detail but overlooked the food intake (only included fortified oils, wheat, and sugar). It is recommended to include information on food group intake and discuss it to justify the nutrient intake inadequacy.

**Response:** Thank you for bringing this to our attention. We have updated the information on food group intake, which now includes Table 4 (Page 9; Line 212-214).

**Comment 14**: The conclusion section includes some recommendations on food fortification rather than dietary diversification or a food-based dietary approach. It is recommended to include some findings on food group intake and make recommendations based on those findings along with fortified oils and/or wheat.

**Response:** We have made the edits to the conclusion section as per your suggestions (Page 16; Line 378-381).

**Comment 15**: Lines #156-157, In case of dietary data, it is recommended to use Goldberg equation to identify outliers rather than just descriptive statistics. Using it will help to achieve more accurate outcomes.

**Response:** Thank you for bringing this to our attention. We also double-checked using the Goldberg equation, and the results were consistent with the descriptive statistics.

**Comment 16**: It is not clear why you compare harmonized average requirement (H-AR) rather than RDA with the nutrient intake. Use of First Quartile (Q1) values rather than median also not clear. Please specify these. Also there is no reference for the harmonized average requirement (H-AR) values.

**Response:** This study seeks to investigate the impact of multi-faceted interventions on mitigating insufficient nutrient intake and nutrient gap in Q1 provide a comprehensive understanding than median (Page 13; Line 308-314).

**Comment 17:** Table #3. Please define the Number in the Initial List & Number in the Updated List in details. As these are not clear from the footnote.

**Response:** Thank you for bringing this to our attention. We have edited Table 3 which is now Table 5.

**Comment 18:** Table #4. Please explain the reason of using p value from the Levene test. Follow this throughout the manuscript where it is applicable

**Response:** Thank you for bringing this to our attention. We have edited the description of Table 4 which is now Table 6 (Page 9; Line 219-228).

**Comment 19**: Table #6. Please specify the meaning of weighted and unweighted Prevalence of Inadequate Intake (percent below H-AR), as nothing is mentioned regarding the sample weight in the manuscript.

**Response**: We have edited Table 6 which is now Table 8.

**Comment 20**: Table #8. The unit of the median value of vitamin A intake should be rechecked

**Response**: We have corrected the unit of the median value of vitamin A intake in Table 8 which is now Table 10.

**Comment 21**: Table #9. it is recommended to use the source (Name of the statistical test) of the p value. Follow this throughout the manuscript where it is applicable.

**Response**: We agreed with your comment and referred to the p-value statistical test in Table 9 which is now Table 11.

**Reviewer #3**

**Comment 1:** Some references are dated (e.g., TDHS 2010). Ensure the use of the most recent and relevant references

**Response:** Thank you for bringing this to our attention. We have revised some references to the most recent one (Page 18; Line 464-489). Furthermore, TDHS 2010 is the most recent reference with nutrition biomarkers in Tanzania. Nutrition biomarkers for TDHS 2022 are not yet available.

**Comment 2**: I do not agree with this information. Maybe “Paucity of information” can be acceptable

**Response:** We have made correction as per your suggestions (Page 4; Line 94).

**Comment 3:** The rationale for excluding refugee camps should be more clearly justified beyond just sample size considerations.

The process for converting local measurements to standard units could be expanded to clarify potential sources of error or variability.

More information on the training of data collectors and quality control measures during data collection would strengthen the methodology.

**Response:** Thank you for bringing this to our attention. We have revised the methodology section as suggested (Page 4-8; Line 111-202).

**Comment 4**: The presentation of results could benefit from a more narrative flow. Consider structuring the results to first describe overall findings, followed by urban-rural comparisons, and then delve into specific nutrients

**Response:** We agreed with the comment and restructured the results section as suggested (Page 8-12; Line 203-282).

**Comment 5**: The discussion could benefit from a clearer connection between the results and the recommendations. For example, specific strategies to improve dietary intake should be linked directly to the deficiencies identified.

Consider discussing potential limitations in greater depth, particularly the impact of seasonality and potential biases in 24-hour dietary recall.

While comparisons to other studies are useful, the discussion should focus more on the unique aspects of the Tanzanian context.

**Response:** Thank you for bringing this to our attention. We have revised the discussion section as suggested to improve the clarity (Page 12-16; Line 283-376).

**Reviewer #4**

**Comment 1**: Abstract:
Acronym WRA should be mentioned on line 31 (first line of abstract) rather than on line 67 (first line of introduction)
Line 51: non lactant should be non-lactating

**Response:** We have reviewed the comment and made the necessary corrections in line with your suggestions.

**Comment 2**: Introduction:
Line 72: Add space between 2021(3)
Lines 82, 84, 85, 102, 119: Use WRA instead of full form
Lines 90, 103: It should be "non lactating" rather than lactating

**Response:** We have reviewed the comment and made the necessary corrections in line with your suggestions.

**Comment 3**: Methods: Sampling procedure was well explained, however additional details needed about the following:
Did the authors use validated questionnaires (adopted from previous study) or were the questionnaires validated? If yes, that should be mentioned. The limitation clearly says information regarding specific brands of oils and wheat flour consumed by women is not collected. This is a big flaw and dilutes the accuracy of the date collection, data analysis and the overall results. This may affect the results of various nutrients especially for nutrient like Vitamin E which is obtained mostly from oils and B-vitamins which are present in wheat flour. How will you justify this?
Line 178-179: Sentence is not making sense. it needs to be rephrased/reworded.
Line 201: The author needs to Define Q1. It should be "Quartile 1(Q1)"

**Response:** The study used a standardized questionnaire that was validated in Ubungo DC, Dar es Salaam (Page 6; Line 142-150). We acknowledge your comments and agree that not specifying certain brands of oils and wheat flour is a limitation of our study (Page 14-15; Line 341-351). Furthermore, we have made all the necessary corrections in accordance with your suggestions.

**Comment 4**: Results
Line 231- Usually SD and IQR is mentioned along with Mean and median respectively. The author has just mentioned the mean and median.
Line 233: Replace Quartile 1 and Quartile 3 with Q1 and Q3
Line 235: p value is not mentioned in the manuscript. Exact p-value should be mentioned.
Exact p values are neither mentioned in tables 4, 5, 6, 7,8,9 nor in the text. It should be mentioned either in text or in table to give the reader a clear picture of the significance.
Lines 240-241, 266, 268, 283, 286, - exact values should be mentioned if not mentioned in the table.
Table 8 only median shown. Add Interquartile range values and exact p-values.
**Response:** Thank you very much for pointing this out and we have made all the necessary corrections in accordance with your suggestions (Page 8-12; Line 203-282).

**Comment 5**: Recent references can be used instead of Ref 24, 30 & 31.
Line 341- WRA can be mentioned rather than full form
Line 347: Remove "and" between Vitamin A, Vitamin B12
Line 396: lowers should be replaced with lower
Line 399: it mentions inadequate intake of Vitamin A can be significantly reduced with the usual intake of oil. Why is the author mentioning only vitamin A and not other vitamin esp Vitamin E, Vitamin D which are now in oils?
Line 402 - NPNL should be used instead of non pregnant, non lactating

**Response:** We have revised the references to the most recent one (Page 18; Line 464-489). Furthermore, we have made all the necessary corrections in accordance with your suggestions.

Sincerely,

Ray Masumo, PhD
