# Supplementary material for: Food and nutrient intake among non-pregnant, non-lactating women of reproductive age of Mbeya in Tanzania: A repeated 24-hour dietary recall
Source: PLOS Glob Public Health. 2024 Dec 5;4(12):e0004010. doi: 10.1371/journal.pgph.0004010 (PMC11620714; doi:10.1371/journal.pgph.0004010)
Supplement: Attachment — Submitted filename: A rebuttal letter_31_10_2024.docx [file pgph.0004010.s004.docx]

Dear Jennifer Tucker, PhD,

Once again, thank you very much for giving us the opportunity to submit a second revised draft of our manuscript titled “**Food and nutrient intake among non-pregnant, non-lactating women of reproductive age of Mbeya in Tanzania: A repeated 24-hour dietary recall**” to *PLOS Global Public Health*.

We appreciate the time and effort that you and reviewers have dedicated to providing valuable feedback on our manuscript.

Here is a point-by-point response to the journal requirement and reviewers’ comments and concerns.

**Journal Requirement**

**Comment 1:** *Please review your reference list to ensure that it is complete and correct. If you have cited papers that have been retracted, please include the rationale for doing so in the manuscript text, or remove these references and replace them with relevant current references. Any changes to the reference list should be mentioned in the rebuttal letter that accompanies your revised manuscript. If you need to cite a retracted article, indicate the article’s retracted status in the References list and also include a citation and full reference for the retraction notice.*

**Response**: We highly appreciate this comment and, we have carefully reviewed the reference list, and ensured it is complete and correct.

**Review Comments to the Author**

**Reviewer #1**

**Comment 1:** *The author provided positive feedback on my questions and suggestions, and the article now reads more clearly as a result. Only one minor comment. Before PLOS Global Public Health decides to accept this article, I would like the author to revise and explain the following issues:
In my previous opinion, I mentioned that "In Food matching, data quality control and validation section, the author used two different food composition tables (TFCT and UFCT)." I think the authors' intention was to enrich the food composition tables, but the authors did not clearly explain this in the article. I would have preferred that the authors state the reason for using two tables in the "Methods" section and explain whether it is appropriate to use data from these two different sources at the same time (Are nutrient measurement methods and units consistent in food ingredient lists from different sources?) instead of writing my original statement directly into the article (Page 7; Line 168-173)..*

**Response:** Thank you very much for pointing this out. We highly appreciate your insightful comment and suggestion. We have rephrased the paragraph as suggested (see page 7; Line 168-175).

Sincerely,

Ray Masumo, PhD
